# Soil and Site Productivity Effects on Above- and Belowground Radiata Pine Carbon Pools at Harvesting Age

**DOI:** 10.3390/plants13243482

**Published:** 2024-12-12

**Authors:** Daniel Bozo, Rafael Rubilar, Otávio Camargo Campoe, Rosa M. Alzamora, Juan Pedro Elissetche, Juan Carlos Valverde, Roberto Pizarro, Matías Pincheira, Juan Carlos Valencia, Claudia Sanhueza

**Affiliations:** 1 Cooperativa de Productividad Forestal, Departamento de Silvicultura, Facultad de Ciencias Forestales, Universidad de Concepción, Concepción 4030555, Chile; dbozo@udec.cl (D.B.); juvalverdeo@udec.cl (J.C.V.); 2 Centro Nacional de Excelencia para la Industria de la Madera (CENAMAD)—ANID BASAL FB210015, Pontificia Universidad Católica de Chile, Santiago 7820436, Chile; ralzamora@udec.cl (R.M.A.); jelisset@udec.cl (J.P.E.); rpizarro@utalca.cl (R.P.); csanhueza@fsplatam.cl (C.S.); 3 Forest Productivity Cooperative, Departamento de Ciências Florestais, Universidade Federal de Lavras, Lavras 37200, MG, Brazil; otavio.campoe@ufla.br; 4 Departamento de Manejo de Bosques y Medio Ambiente, Facultad de Ciencias Forestales, Universidad de Concepción, Concepción 4030000, Chile; 5 UNESCO Chair Surface Hydrology, University of Talca, Talca 3467769, Chile; 6 Instituto Interdisciplinario Para la Innovación, Universidad de Talca, Talca 3467769, Chile; 7 Facultad de Ciencias Forestales y de la Conservación de la Naturaleza, Universidad de Chile, Santiago 8820808, Chile; 8 Forestal Mininco S.A., Avenida Alemania 751, Los Ángeles 4440000, Chile

**Keywords:** *Pinus radiata*, carbon stock, biomass, forest floor, soil organic carbon

## Abstract

*Pinus radiata* D. Don is the most widely planted forest species in Chile, making it crucial to understand carbon pools in adult plantations. This study aimed to evaluate the effect of soil type and site productivity on the total carbon stock in adult radiata pine plantations, considering sites with contrasting water and nutrient availability. We selected 10 sites with sandy and recent volcanic ash soils, representing a productivity gradient. At each site, three 1000 m^2^ plots were established to quantify the carbon stock of total biomass using allometric equations and in situ carbon assessments of the forest floor and mineral soil (up to 1 m deep). The results indicated significantly higher carbon stocks in the mineral soil of recent ash sites (281.4 Mg ha⁻^1^) compared to sandy soils (139.9 Mg ha⁻^1^). The total site carbon was also higher in recent ash (473.2 Mg ha⁻^1^) than in sandy sites (330.9 Mg ha⁻^1^). A significant relationship was found between stand productivity and soil organic carbon (r^2^ = 0.88), as well as total carbon stock (r^2^ = 0.91) when considering soil type. These findings highlight the importance of including assessments up to 1 m depth and developing soil type and productivity models to improve site carbon stock estimates.

## 1. Introduction

In the last century, atmospheric carbon dioxide (CO_2_) concentrations have increased by 10 to 20% due to anthropogenic activities [1]. Greenhouse gas emissions have triggered severe environmental changes including increased temperatures and intense and prolonged droughts worldwide [2]. Strategies to reduce emissions and promote carbon sequestration consider forest plantations as a short-term option for carbon (C) capture [3,4]. This approach may also improve ecosystem services such as hydrological cycle regulation, reducing desertification, and providing wood and fiber as an action to mitigate climate change [5].

Given the importance of forests in mitigating climate change, a correct understanding and estimation of carbon stocks across ecosystems is required. Carbon accumulation in forest plantations is strongly linked to species, age, and management [2,6,7], but also to abiotic factors (e.g., climate and soil) [8,9,10,11,12]. Studies have estimated that global forest resources accumulate between 861 and 899 Pg C, where 44% is distributed in soils (up to 1 m of depth), 42% in living above- and belowground biomass, 8% in deadwood, and 5% in litterfall [3,13,14]. The soil organic matter (SOM) is a key component of soil, which contains more organic carbon than global vegetation and the atmosphere combined [15]. A large part of SOM is constituted by humic substances, which are key to the stabilization and mineralization of SOM to CO_2_ [16]. These substances have key physical and chemical functions in soil, like a high cation exchange capacity, a high water-holding capacity, and the abilities to adsorb or complex cationic nutrients, immobilize heavy metals, and affect the availability of phosphorus in soil [17]. According to Gerke [17], the stability of humic substances is based on the resistance to attack by microorganisms (due to their aromatic cores) and the reaction of the soil humic substances with mineral surfaces (reducing microbial degradation). This allows carbon recalcitrance, leaving its aggregates protected and stabilized for longer in the mineral soil.

Most studies worldwide provide precise and detailed aboveground carbon estimates. However, carbon accumulation in forest residues, forest floors, and mineral soil may represent more than 60% of the global carbon reserves in forests [3], which demonstrates the importance of including these components in the study of carbon estimations. In fact, the highest proportion of organic carbon pools is found in mineral soil (up to 1 m deep) [3,18], in particular in the first 10 cm of the soil, and where the first 40 cm accumulate more than 50% of the total soil carbon [2,19]. Soil carbon from fallen leaf litter and underground biomass decomposition [20] increases over time with the age of the stand [2,21] until it reaches a maximum defined by the soil and site dynamics if no disturbance exists. In this regard, soil type is a key factor in the accumulation of carbon in depth [13]. Clay soils retain organic matter within their aggregates, slowing down their decomposition, which promotes long-term carbon accumulation in the soil [22]. Sandy soils in turn have larger pores, retain less moisture and nutrients, and have faster rates of organic matter decomposition. These factors reduce tree and stand growth but also the potential accumulation of carbon in the soil [8,23,24]. Soil texture affects soil fertility and soil moisture together, both key factors defining site productivity and carbon sequestration within a given climate [13,25].

Tree above- and belowground biomass is the second-largest C stock in forest ecosystems [2,3]. Stem biomass is the largest carbon sink, and as trees age, carbon accumulation increases [2]. Similarly, but less known, belowground biomass becomes the major source of soil organic matter as it decomposes over time [26]. However, alive root biomass estimates are usually limited and uncertain for most ecosystems, and constant generalized estimates as a fraction of aboveground biomass have been proposed for accounting for this pool [27].

Another important carbon pool in forest ecosystems is the forest floor, a layer of dead and decomposing plant material on top of the mineral soil, which includes accumulated leaf litter, woody material, and fruits in varied degrees of decomposition [28]. Although this pool usually represents a lower proportion of all aboveground carbon in forests, it is estimated that it may represent around 5% of all aboveground biomass [3], and play a key role in the contribution of organic matter and nutrients to the mineral soil from aerial biomass [9,26].

*Pinus radiata* D. Don (radiata pine) is one of the most important fast-growing pine species cultivated in the southern hemisphere, mainly in New Zealand, Chile, Australia, and South Africa [29,30]. Plantations are among the most productive species, and their high productivity depends on advanced genotypes, silviculture, and environmental conditions [30,31,32,33,34]. In Chile, *P. radiata* represents the most important forest species with more than 1.3 million ha of the approximately 2.3 million ha of total commercial planted forests [35]. According to the Council of Ministers of Chile [36], the country has committed to being carbon neutral by 2050; therefore, optimizing the land planted with fast-growing species is one the most effective and efficient ways to achieve this commitment [37].

Olmedo et al. [8] evaluated radiata pine plantations’ C accumulation in Chile and showed that plantations with the highest C stock were found in sedimentary and volcanic ash soils with high water resource availability. Contrastingly, water-restricted sites of lacustrine and sandy soils showed the lowest C stocks. Carbon stock estimates of radiata pine plantations require soil and productivity information for the development of models that may be able to accurately estimate above- and belowground biomass carbon and mineral soil and forest floor pools. Our objective was to develop and evaluate specific versus generalized soil site-productivity-based C stock estimation models estimating biomass, mineral soil, and forest floor C pools of intensively managed radiata pine plantations at harvesting age at contrasting soil sites.

## 2. Results

### 2.1. Stand Growth Metrics and Carbon Stocks of Above- and Belowground Biomass

Recent ash soil sites presented a higher diameter at breast height (DBH) and total height (HT) than sandy sites (*p* < 0.05). The average individual tree DBH was 36.7 cm for recent ash soil sites vs. 34.1 cm in diameter for sandy soil sites. Similarly, recent ash sites showed a higher HT of 33.6 compared to 30.5 m for sandy sites (Table 1). Stocking (NHA) was the only variable where sandy sites showed larger values than recent ash sites (492 vs. 399 trees ha^−1^). No differences in basal area (BA), stand volume (VHA), or mean annual increment (MAI) were observed between sandy and recent ash sites (*p* > 0.05) (Table 1).

**Figure 1:**
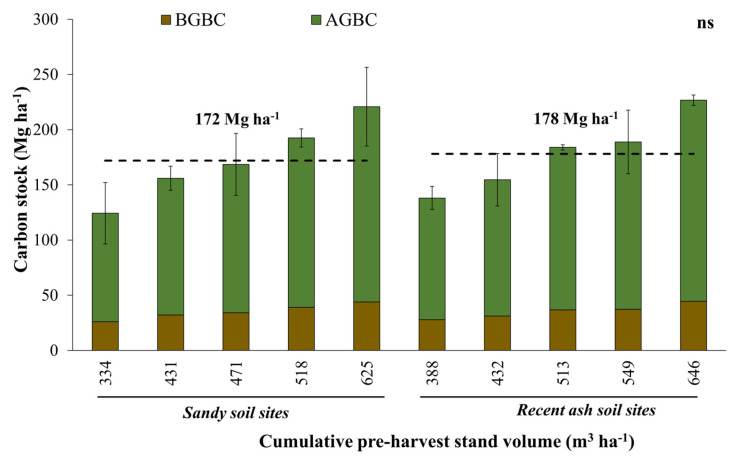
Aboveground biomass carbon stock (AGBC) and belowground biomass carbon stock (BGBC) at each site by soil type and cumulative over bark stand volume at harvesting age. The dashed lines represent the average total biomass carbon stock for sandy and recent volcanic ash soil sites. Error bars indicate standard deviations for each site (n = 3).

No significant differences were observed on average for total biomass carbon stock (*p* = 0.66), or for aboveground (*p* = 0.62) or belowground (*p* = 0.87) estimates between sandy and recent ash sites. However, a slightly higher total biomass carbon stock was observed in recent ash sites (178.5 Mg ha^−1^) compared to sandy sites (172.4 Mg ha^−1^) (Figure 1 and Table 2).

A direct relationship between site productivity and carbon stock in total biomass was observed, which suggested a positive effect of site productivity on carbon stock accumulation (*p* < 0.05) (Figure 1).

### 2.2. Organic Carbon Stock in the Mineral Soil (Up to 1 m Deep)

**Figure 2:**
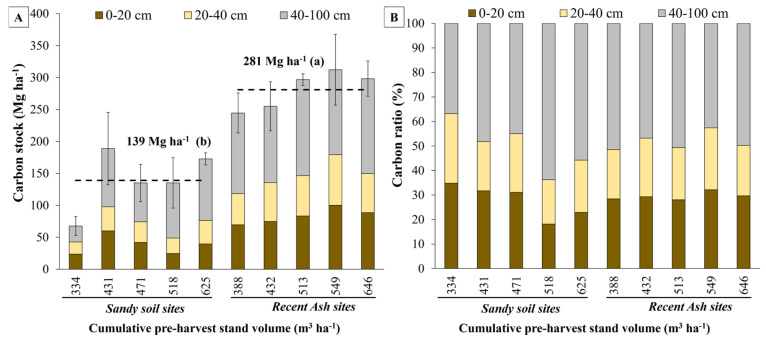
(**A**) Soil carbon stock up to 1 m depth; and (**B**) proportion of carbon for each soil depth, at each site by soil type and cumulative over bark stand volume at harvesting age. The dashed lines represent the average values of soil organic carbon stock for sandy and recent volcanic ash soil sites. In (**A**), differences between soil type means are indicated by lowercase letters and error bars represent standard deviations for each site (n = 3).

Recent ash soils presented a higher organic carbon stock in the mineral soil than sandy soils (*p* < 0.05) (Figure 2A and Table 2) and showed 29.5% of the organic carbon stock in the mineral soil accumulated at 0–20 cm depth, 22.2% at 20–40 cm depth, and the remaining 48.2% accumulated between 40 and 100 cm depth (Figure 2B). Similarly, sandy sites accumulated 27.7 and 22.4% at 0–20 and 20–40 cm depth, respectively, and the remaining 49.9% at 40–100 cm depth (Figure 2B).

The LR site (the least productive sandy soil), according to observations of carbon stock in total biomass, had the lowest organic carbon stock in the mineral soil up to 1 m depth (67.7 Mg ha^−1^) and showed significant differences from most other higher productivity sites (*p* < 0.05) (Table 2 and Figure 2A). The RA site (the most productive recent ash soil) had the highest organic carbon stock in the mineral soil (312.3 Mg ha^−1^), showed the second-highest volume (549.1 m^3^ ha^−1^), and was significantly higher than most other sites (*p* < 0.05) (Table 2 and Figure 2A).

**Figure 3:**
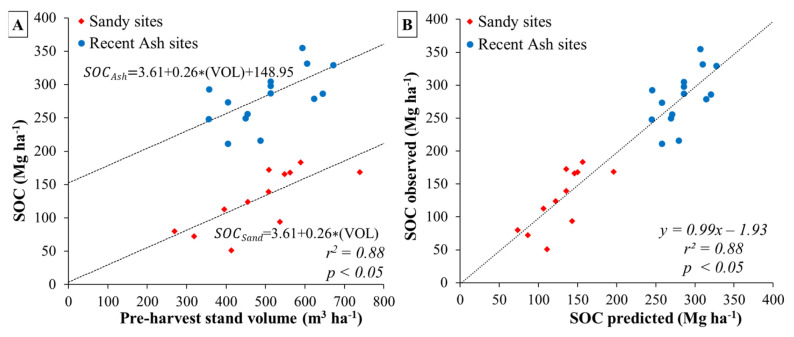
(**A**) Relationship between site productivity (cumulative over bark stand volume at harvesting age, VOL) and organic carbon stock in the mineral soil (up to 1 m deep) (SOC) using soil type as the dummy variable; and (**B**) relationship between observed SOC and predicted SOC estimated using the dummy variable regression adjusted model.

A positive relationship was observed between site productivity and organic carbon stock in the mineral soil (up to 1 m deep), suggesting that an increase in organic carbon in the mineral soil could be explained by site over bark stand volume at harvesting age for each soil type (Figure 2A and Figure 3A, Table 2). Regression analysis of productivity against organic carbon stock in the mineral soil (up to 1 m deep) for all selected soil sites showed a significant but weak relationship between both variables (*p* < 0.05, r^2^ = 0.16) (Table 3). However, when considering soil type as a dummy variable in the regression model, the coefficient of determination of the model increased up to r^2^ = 0.88 (*p* < 0.001). The adjusted model had the following form: Soil organic carbon = 3.61 + 0.26 × (VOL) + 148.45 × (0 = Sand or 1 = Ash) (Table 3, Figure 3A,B). This model suggests that although the regression lines for sand and ash soil types have the same slope, they differ in the intercept, indicating a strong effect of soil type on organic carbon stock in the mineral soil (an increase of 148.9 Mg ha^−1^ was observed for recent ash soils compared to sandy soils).

### 2.3. Forest Floor Carbon Results

**Figure 4:**
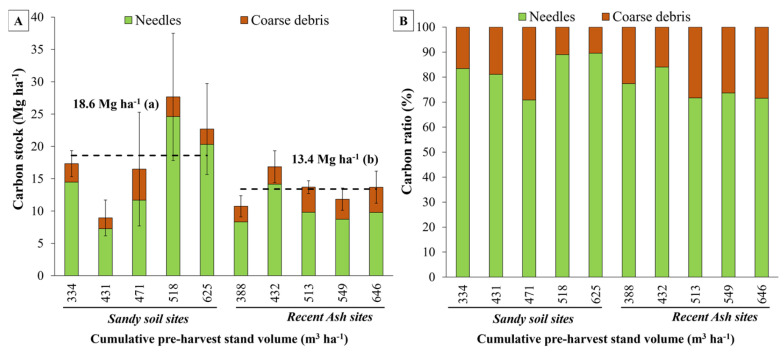
(**A**) Forest floor carbon stock in organic horizons and coarse woody debris; and (**B**) proportion of carbon stock in forest floor and coarse woody debris at each site by soil type and cumulative over bark stand volume at harvesting age. The dashed lines represent the average value of forest floor carbon stock for sandy and recent volcanic ash soil sites. In (**A**), differences between soil type means are indicated by lowercase letters and error bars represent standard deviations for each site (n = 3).

Sandy soil sites presented higher forest floor carbon stock than recent ash soils (*p* < 0.05) (Figure 4A and Table 2). On average, in sandy soil sites, approximately 82.8% of the forest floor carbon is found in organic horizons, while only 17.2% is attributed to coarse woody debris (Figure 4B). In contrast, recent ash soil sites show that 75.6% of the forest floor carbon stock accumulated in organic horizons and 24.4% in coarse woody debris (Figure 4B). No significant correlation was discovered between site productivity and carbon accumulation in the forest floor carbon pool (*p* > 0.05). Additionally, adjusted models did not reveal any effect of soil type on this relationship (Table 2).

### 2.4. Total Carbon Stock (TCS) Results

**Figure 5:**
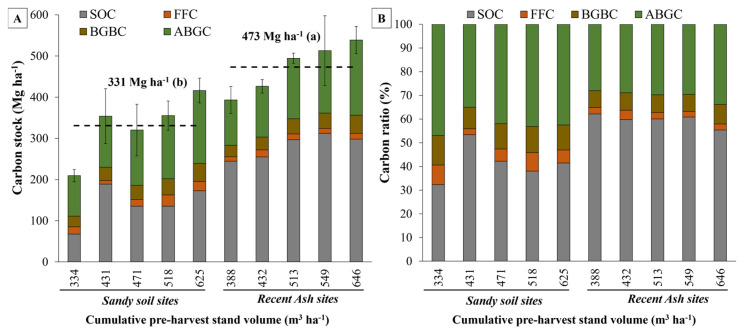
(**A**) Total carbon stock for soil organic carbon (SOC), forest floor carbon (FFC), belowground biomass (BGBC), and aboveground biomass (ABGC); and (**B**) proportion of total site carbon stock at each site by soil type and cumulative over bark stand volume at harvesting age. The dashed lines represent the average value of total carbon stock for sandy and recent volcanic ash soil sites. In (**A**), differences between soil type means are indicated by lowercase letters and error bars represent standard deviations for each site (n = 3).

Recent ash soil sites exhibited 59.6% of the total carbon stock (TCS) stored in the soil organic carbon (SOC) stock, which was measured up to a depth of 1 m, amounting to 281.4 Mg ha^−1^. Following closely was the carbon stock accumulated in the total biomass, constituting 37.5% (178.5 Mg ha^−1^). The forest floor pool, accounting for 2.9%, represented the lowest carbon stock for these soils (13.4 Mg ha^−1^) (Figure 5A,B and Table 2). Conversely, for the sandy soil sites, the highest proportion of TCS was found in total biomass with 52.7% (172.4 Mg ha^−1^) of the TCS, followed by the SOC with 41.5% (139.9 Mg ha^−1^). Once more, the forest floor exhibited the lowest carbon stock, amounting to 5.8% (18.6 Mg ha⁻^1^), with sandy soils showing a higher carbon stock in this pool compared to recent volcanic ash soils (Figure 5A,B and Table 2).

For sandy soils, the LR site, the site with the lowest productivity, showed the lowest total carbon stock (209.4 Mg ha^−1^) and showed significant differences with all other sandy sites, except with the MI site (Figure 5A and Table 2). For the recent ash soils, the ST site with the highest productivity, had the highest total carbon stock (538.7 Mg ha^−1^), showing a significant difference with the lowest productivity ash site HI (393.4 Mg ha^−1^) (Table 2 and Figure 5A).

**Figure 6:**
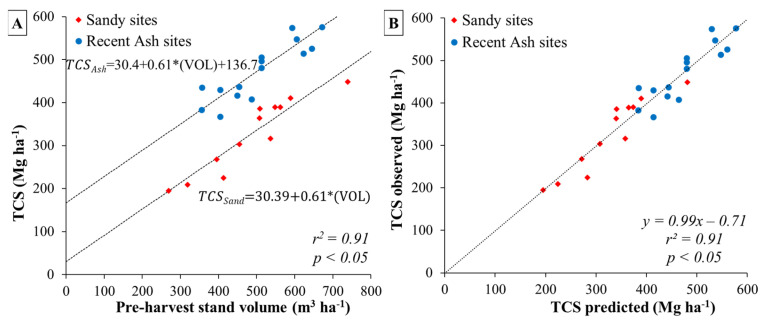
(**A**) Regression models of the relationship between cumulative over bark stand volume at harvesting age (VOL) and total site carbon stock (TCS), considering soil type as a dummy variable; and (**B**) relationship between observed and predicted TCS using the dummy variable model.

Similar to what was observed for the SOC pool, a positive relationship was observed between stand productivity (cumulative over bark stand volume at harvesting age) and total site carbon stock (TCS), which suggests that TCS increases as stand volume increases across each soil type (Figure 5A and Figure 6A, Table 2). Regression analysis between stand volume and TCS showed a significant relationship (*p* < 0.05, r^2^ = 0.49) (Table 4). When considering soil type as a dummy variable in the regression model, the coefficient of determination increased up to r^2^ = 0.91 (*p* < 0.001, Figure 6A,B). The regression model considering soil type as a dummy variable had the following form: TCS = 30.39 + 0.61 × (VOL) + 136.66 × (0 = Sand or 1 = Ash) (*p* < 0.05) (Table 4). The dummy variable model showed the same slope (0.61) but differed in the intercept parameter, which was affected by the soil type dummy variable (with an increase of 136.66 Mg ha^−1^ for the ash soil type). Therefore, for the same level of site productivity, the recent ash sites presented a higher total carbon stock due to soil type, with a positive offset similar to the mean value difference observed between sandy (330.9 Mg ha^−1^) and recent ash (473.2 Mg ha^−1^) sites (Figure 5A).

## 3. Discussion

### 3.1. Carbon Stock in Above- and Belowground Biomass

Mean carbon stocks in above- and belowground biomass (total biomass) showed no significant differences between the sandy and recent volcanic ash soil types. Similarly, no differences were observed in average stand volume, MAI, basal area, or carbon stock in biomass pools between soil types, but important differences were observed in average DBH and HT (Table 1 and Table 5). On average, larger trees were observed in recent volcanic ash sites compared to the sandy sites (Table 1). However, higher stockings were observed in sandy soils, which on average showed 93 trees ha^−1^ more than volcanic ash sites. Differences in stocking obey to differences in operational management schemes of these stands where recent volcanic ash sites are targeted to generate larger diameters for sawn timber objectives [38] compared to the multipurpose (pulp and structural sawtimber) nature of sandy sites. For this reason, although there were no differences in mean C stock in total biomass at stand level between sandy and recent ash soil types, differences were observed at the individual tree level in aboveground biomass, with trees having greater individual C stock in recent ash soil sites (0.74 Mg tree^−1^), increasing the C stock at the individual tree level with respect to sandy soil sites by 25.5% (0.59 Mg tree^−1^). Other studies that have evaluated long-term productivity in radiata pine stands in Chile have also shown higher growth in diameter, height, and stand volume in recent ash and red clay soil sites compared to sandy soil sites [32,34].

Our results for total biomass C stock of radiata pine stands under intensive management were similar to those reported by Olmedo et al. [8], where for similar age and silviculture stands, there were slightly higher values of biomass C stock with 181.2 to 214.0 Mg ha^−1^ for sandy and recent volcanic ash soils types, respectively. Differently, in Argentina, Ferrere and Lupi [42] reported only 105.2 Mg ha^−1^ of C stock in the total biomass in 21-year-old radiata pine stands, which is lower than the values reported by Olmedo et al. [8] and for our study. Thus, although the study of Ferrere and Lupi [42] was in stands of similar age (21 years) and standing volume (390.5 m^3^ ha^−1^), its higher stocking (870 trees ha^−1^) resulted in smaller DBH (27.5 cm) and HT (18.2 m) values compared to the stands of our study.

In the case of sandy soil sites, the highest proportion of carbon stock was found in total biomass (52.1%) which is comparable with the value reported by Olmedo et al. [8] (70.5%). The differences in carbon composition among stand components in the two studies primarily stem from the depth at which soil carbon pools were assessed. Our study evaluated soil carbon stocks up to a depth of 1 m, whereas Olmedo’s study measured soil carbon stocks only up to a depth of 30 cm. However, in considering up to 40 cm depth, our results are quite comparable to those reported by Olmedo et al. [8]. In fact, the C in the total biomass, considering a 40 cm depth, would have reached 66.7% of the total site C stock. In the case of recent volcanic ash sites, the C stock in the total biomass decreased significantly with respect to sandy soils, corresponding to 37.7%, and being the second-largest C reservoir in order of importance after SOC. In the study of Olmedo et al. [8], the proportion of C in the total biomass for recent ash soils was 64.3%, much higher than our value. Again, this difference in C proportions with our study was due to the different depths of soil sampling, and if we consider only up to 40 cm depth, the proportion of C in the total biomass increases to 52.8%, being even lower than the value estimated by Olmedo et al. [8].

A direct relationship between stand volume and total biomass C stock was observed independent of soil type. Balboa-Murias et al. [43] found an increase in aboveground biomass C with an increase in site index, similar to what we observed with stand productivity. They found also that thinning increased tree growth and C accumulation in stems, and reported, similar to our results, between 96.0 and 187.0 Mg ha^−1^ of C stock in the aboveground biomass for 30-year-old plantations. While, in a global assessment of the effect of thinning on C accumulation in forests, Zhang et al. [44] found that overall thinning significantly increased total tree aboveground biomass C stock (23.9%); however, belowground biomass C stock remained unchanged, with a positive effect also in an increase in the mean DBH by 18.9%, showing a positive effect of intensive plantation management on C stock.

### 3.2. Organic Carbon Stock in the Mineral Soil (Up to 1 m Deep)

Soil organic carbon concentrations were higher in volcanic ash soils than in sandy soils along the soil profiles (Table 6). Large differences between these soil types may be explained by soil texture and its effect on organic matter protection and accumulation over time [13,22]. For both sites, carbon concentration decreased in depth (Table 6), similar to what is reported in many studies [2,19,42], and as expected due to soil matter organic accumulation from root decomposition and forest floor inputs [20,26].

For C stock in the mineral soil, Olmedo et al. [8] reported on average 68.7 to 109.7 Mg ha^−1^ for sandy and volcanic ash soil types, respectively. In our study, due to our deeper sampling up to 1 m in depth, we found 139.9 and 281.4 Mg ha^−1^ for the same soil types, respectively. When considering our SOC values only up to 40 cm depth, to resemble the depth measured by Olmedo et al. [8], the mean carbon stock values for sandy soils were very similar between both studies, with 68.7 Mg ha^−1^ vs. 68.1 Mg ha^−1^ (our study). In sandy soils, when considering only up to 40 cm, the SOC represented around 26% of the total carbon stock of the stand; however, when considering up to 1 m, it increased to 42.3%.

In another recent study carried out by Crovo et al. [45] in central Chile, the C stock was compared between native forest and radiata pine plantations across five soil types, of which one site corresponded to Andisols, with characteristics similar to the recent ash soil sites that we evaluated in our study. In this study, the Andisol site presented the highest SOC stock, without significant differences between forest types, with a SOC value accumulated up to 120 cm depth for the native forest of 246.6 Mg ha^−1^ and for pine plantation of 243.7 Mg ha^−1^, with very similar values to the mean SOC stock up to 1 m depth for the recent ash soil sites found in our study (281.4 Mg ha^−1^).

Interestingly, in the case of recent volcanic ash soils, when considering SOC only up to 40 cm, we found 146.0 Mg ha^−1^ of C in mineral soil, much higher than the values reported by Olmedo et al. [8] up to 30 cm depth (109.7 Mg ha^−1^), similar to what Crovo et al. [45] reported for the same depth for the radiate pine plantation (129.6 Mg ha^−1^), but lower than what Ferrere and Lupi [42] reported for radiata pine stands in Argentina (210.6 Mg ha^−1^ of C in mineral soil up to 50 cm depth). In our study, in recent volcanic ash soils, SOC represented on average 59.5% of the total site C stock. When considering only a 40 cm depth, it represented 43.2%, a higher proportion than Olmedo et al. [8], who reported only 33.0%, and a smaller proportion than what was reported by Ferrere and Lupi [42] in Argentina where it accounted for 64.9% of the total site carbon. For recent volcanic ash soils, at the depth of 60 to 100 cm, we found 135.4 Mg ha^−1^ of C, which, like in sandy soils, suggests that a large unaccounted amount of carbon at depth needs to be considered for the correct assessment of C sequestration estimates from forest plantations.

Our results emphasize what was indicated by Houghton [46] for terrestrial ecosystems, indicating that two to three times more carbon is stored in the top meter of soils than in the living biomass. Among the soil factors that drive the C stock in soils are the parent material and soil properties, such as the aggregation of its particles, the clay and silt content, and the mineralogy of the clays and their specific surface area [12,13,47], which display different capacities for soil organic matter (SOM) stabilization and protection [48,49].

Recent volcanic ash soils (Andisols) present secondary minerals, including short-range-order (SRO) minerals [12], such as allophane and imogolite [50]. These clay type and soil aggregates, with high specific surface area and variable charge, provided physical and chemical protection and stabilization for SOM [12,49,50], resulting in higher C stocks than sandy soil sites. Our results and those of Olmedo et al. [8] and Crovo et al. [45] suggest that a high amount of C stock is stored and maintained in volcanic ash soils, due to clay type and the higher clay and silt content, accumulating more soil organic matter [13,47,51,52]. Therefore, the content of clays of the SRO mineral type, such as allophane and imogolite in recent volcanic ash soils, allows Andisols to accumulate one of the largest SOC stocks in the temperate forest [12,47,53].

In addition, sites with finer soil textures and greater water retention capacity, such as recent volcanic ash soil sites (Table 6), also show high microbial activity that increases the rate of decomposition of organic matter in fine roots and its input into the soil [22,26,47,54]. Sandy soils, having 141.5 Mg ha^−1^ less C than volcanic ash soils, have a lower potential to sustain larger amounts of soil carbon because of their coarse texture, larger pore size, and lower water and nutrient availability that affects stand productivity but also the storage and speed of decomposition of organic matter [8,23,24,55,56].

### 3.3. Forest Floor Carbon Stock

The lowest C stock in our study was found on the forest floor, and significant differences were observed between soil types. Sandy soils showed higher estimates (18.6 Mg ha^−1^) compared to recent volcanic ash soils (13.4 Mg ha^−1^).

Our results were similar to estimates reported by Oliver et al. [57] in New Zealand for 15- and 16-year-old *Pinus radiata* stands, where forest floor soil C accumulation ranged between 12.4 and 16.7 Mg ha^−1^. However, in Spain, a mean of 14.8 Mg ha^−1^ of C in the forest floor was found for radiata pine stands, but with a range of 2.5 to 42.1 Mg ha^−1^ across sites [9]. Our values were higher than those reported by Olmedo et al. [8], with 7.1 and 9.1 Mg ha^−1^ for sandy and volcanic ash soil types, respectively; and those reported by Ferrere and Lupi [42] in Argentina with 8.3 Mg ha^−1^. In our study, C stock in the forest floor represented the lowest C stock in the stand, reaching a 5.6% average at sandy and a 2.8% average for recent volcanic ash soil types. The forest floor, despite being the smallest C reservoir, represents an important contribution to mineral soil C but also has a key role as a nutrient reservoir for successive rotations [9,26].

**Figure 7:**
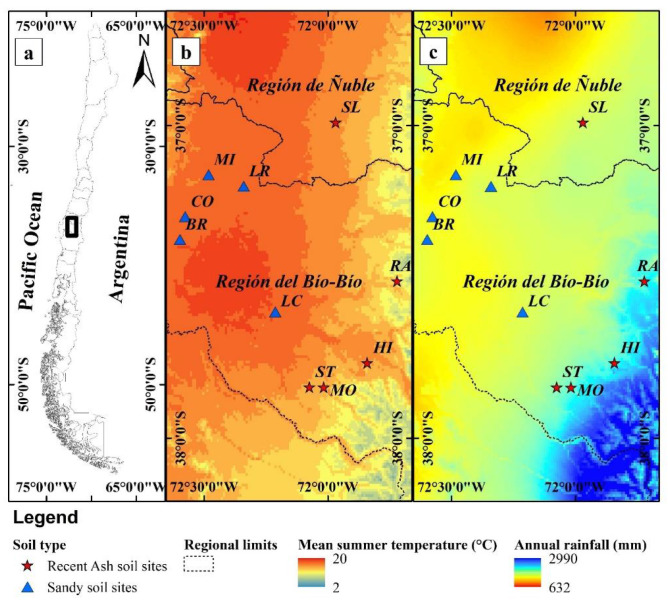
(**a**) Study area in Central South Chile, (**b**) Study area and site locations’ mean summer (January to March) temperature (°C), (**c**) Study area and site locations’ mean annual rainfall (mm yr^−1^) (site code name is presented in Table 5).

In our study, the effect of soil type on the amount of C in the forest floor was important but not site productivity. Also, similar to what was found by Petritan et al. [58], no correlation was observed between stand volume and C stock in dead wood residue accumulation. In a previous study by Cartes-Rodríguez et al. [59] in Chile in *Pinus radiata* plantations at harvesting age, biomass residues for contrasting soil types were evaluated and, similar to our results, a larger proportion of residues was observed in sandy compared to recent volcanic ash soil types. In the previously indicated study, 34.2 Mg ha^−1^ of residues were estimated for sandy sites and 27.3 Mg ha^−1^ for volcanic ash soil sites. Applying a simplified C conversion factor (C = 50% organic matter), the C average value matches our results. The greater C stock in the forest floor observed for sandy compared to recent volcanic ash soil sites may be because sandy sites have higher temperatures and lower humidity (Figure 7, Table 5), which could slow down decomposition rates of organic matter, as forest floor carbon stock is affected by precipitation, altitude, crown cover, and water deficit [9,10,26].

### 3.4. Total Carbon Stock

Large carbon stocks were found in radiata pine plantations evaluated for sandy and recent volcanic ash sites. The observed values, similar to what has been reported in previous studies, indicate that most productive sites that have less water and nutritional limitations reach the largest total carbon storage capacity [8,60].

Our estimated values were higher than those reported by Olmedo et al. [8] in a recent study carried out in Chile in which carbon stocks were evaluated for radiata pine plantations considering different soil site types. In that study, for the same soil types and plantations of similar age, total carbon stock mean values ranged from 257.0 to 332.8 Mg ha^−1^ for sandy and volcanic ash, respectively. However, Olmedo et al. [8] only estimated SOC until 30 cm of depth, resulting in a underestimation of total site carbon stock with respect to our study. In other recent study, Ferrere and Lupi [42] evaluated the C stock of radiata pine stands in Argentina, finding an average of 324.1 Mg ha^−1^ of C in stands of similar ages. However, they only measured SOC up to 50 cm in depth, so again an important underestimation of total site carbon that is accumulated in forest plantations. These results indicate the importance of measuring and accounting for soil carbon in depth to correctly evaluate total site carbon pools. In fact, our results of C stock in volcanic ash only up to 40 cm depth (337.9 Mg ha^−1^ of C) account for similar values to both previous studies with soils of similar characteristics.

### 3.5. Relation Between C Stocks and Stand Productivity

We found a positive linear relationship between over bark stand volume and total site C stock (soil organic carbon + carbon in the total biomass + carbon in the forest floor) (*p* < 0.05). This relationship improved significantly when models were adjusted considering soil type as a dummy variable (*p* < 0.001), providing independent regressions for each soil type. Interestingly, regardless of soil type, there is an increase in SOC and total C stock that depends on stand volume, and the same slopes for sandy and volcanic ash soil types of 0.26 for the C in the mineral soil and 0.61 for C stock of all stand components suggest that site productivity, as an expression of site resource availability, has a close relationship with C accumulation.

Soil type influenced the intercept of the model, with an increase of 148.9 Mg ha^−1^ for SOC for recent volcanic ash soil compared to sandy soil sites (Figure 3A, Table 3); and with an increase of 136.7 Mg ha^−1^ for total site carbon stock in recent volcanic ash compared to sandy soils (Figure 5A, Table 4). The increase in C stock found in the soil aligned with the level of productivity of the site and soil type. This also may be related to the source of organic matter entering the soil, coming mainly from the decomposition of fine roots and litterfall [26,61]. Therefore, sites with higher stand volume will have a greater contribution of organic matter from the decomposition of the roots and forest soil, providing C to the mineral soil.

This study provides interesting equations to estimate C stocks in mineral soils and total C stock at the harvesting age of radiata pine stands for sandy and recent volcanic ash soils for radiata pine plantations. Few studies have evaluated relationships between stand productivity and site C stocks. Kranabetter [60] showed a slight trend of an increase in mineral soil C (until 50 cm depth) along a productivity gradient in Canadian boreal forests. However, the trend was significant when considering all above- and belowground stand components (C in the mineral soil + forest soil + residues + total biomass) and stand height. Pinto et al. [56], investigating *Pinus taeda* plantations in Brazil, related soil attributes to site productivity. They found a positive relationship between stand mean annual increment (MAI) with clay content and with soil C stocks. However, another study that related C stock to site productivity, developed by van den Bor et al. [62], evaluating *Pinus halepensis* plantations and *Quercus* native forest in central Spain, found a significant relationship between basal area and total C stock of stands, and for individual aboveground and belowground biomass and litter components. However, they did not find a relationship between basal areas and C stock in the mineral soil for any of the investigated species.

Since not all the studies found a significant trend between C stock in the mineral soil and site productivity, this study provides a significant linear model (*p* < 0.05; r^2^ = 0.88) and suggests that productivity could be used to estimate site and soil C stocks. Given that our study showed that C stock in the mineral soil in depth is a large proportion of the total site carbon stock, which is rarely considered in C stock calculations, it emphasizes its importance for future assessments and should be incorporated in carbon sequestration models. Our models improve current C stock estimates for radiata pine plantations at harvesting age and emphasize the importance of developing soil-type-specific models to appropriately estimate site C stock.

We recognize some limitations in our study and recommend cautious use of the developed models. Our models are constrained to stands close to harvesting age and may lose accuracy if applied to younger stands of the same productivities. Similarly, our models were developed for specific soil parent materials and use in other soils may not provide accurate estimates of soil carbon. Given the sensitivity of soil carbon differences among soil types, we also suggest using our models within a similar range of soil physical properties observed in our study.

## 4. Materials and Methods

### 4.1. Study Area

Sites were selected representing a productivity gradient of *Pinus radiata* plantations (15.2 to 28.1 m^3^ ha^−1^ yr^−1^) under intensive management at harvesting age (20–23 years old, the age at which radiata pine plantations are harvested in Chile), covering a part of the productive area of radiata pine plantations in Chile (Table 5). The sites were located in the Central Valley and the Foothills of the Andes in central and south-central Chile and spread from the Ñuble (36°59′ S) to the Biobío region (37°50′ S), and west to east from 72°35′ W to 71°43′ W. Altitudes ranged from 106 to 849 m above sea level and soils considered five stands located in coarse volcanic sands and five stands were in recent volcanic ash soil sites [40,41] (Figure 7). Selected stands at each soil site condition were planted between 1999 and 2001 with initial planting density ranging from 1000 to 1250 trees ha^−1^.

All sites were established with soil preparations considering a router at 60 cm deep plus disking. Recent volcanic ash soils received traditional operational fertilization considering 100 to 150 g per plant of NPK mix (10 to 15 g N + 10.9 to 13.2 g P + 8.3 to 12.5 g K) plus 2 to 3 g per plant of boron applied after planting. For sandy soil sites only, boron was applied. Long-term studies have not demonstrated growth gains due to fertilization [34]. Fundamentally, operational fertilization at these sites aims to support establishment and cover boron needs as a critical element affecting normal plantation development [63]. Operational weed control of stands considered the pre-planting total area and 2 years banded weed control after planting in most conditions. All selected stands were pruned at least to 5.5 m height and thinned with 1 or 2 commercial thinning practices according to the silvicultural program applied to the stand of each site. Final stand stockings at harvesting reached 400 to 500 trees ha^−1^.

### 4.2. Data Collection and Sampling

Inventory measurements and samplings were carried out between March and May 2022. At each site, in order to capture stand variability, three 1000 m^2^ inventory plots were established to measure diameter at breast height (DBH, cm at 1.3 m height) and total height (H, m) for all trees, and the forest floor and mineral soil was sampled within each inventory plot. A total of 30 final plots (15 for sandy and 15 for recent volcanic) were measured considering all site locations.

### 4.3. Volume and Carbon Stocks of Above- and Belowground Biomass Estimations

Published and local allometric equations, developed for radiata pine plantations [64,65], were used to estimate individual tree aboveground (AGB) and belowground (BGB) biomass carbon stock values. With individual tree inventory measurements from each plot considering the number of trees, DBH and HT were used to estimate tree over bark volume, adding tree volume estimates at a plot level and scale estimates to a hectare level. Calculations considered an individual tree local volume equation (V) developed by CMPC Forestal Mininco Forest Company [66]:

(1) $$V=-0.00214+0.0000295*D^{2}+0.001349*H+0.00002486*D^{2}*H$$

where V = individual tree over bark volume (m^3^ tree^−1^), D = DBH (cm), H = height (m).

The aboveground biomass of each component (stem, bark, branch, and needle) of the trees was calculated using the following published [65] allometric equations (Equations (2)–(5)) developed for radiata pine:

(2) $${AGB}_{stem}=0.02389*{{(D}^{2}*H)}^{0.93216}$$

(3) $${AGB}_{bark}=0.00127*{{(D}^{2}*H)}^{0.99646}$$

(4) $${AGB}_{branch}=0.00431*{{(D}^{2}*H)}^{0.92709}$$

(5) $${AGB}_{needles}=0.19428*{{(D}^{2}*H)}^{0.48666}$$

where AGB_stem_, AGB_bark_, AGB_branch_, and AGB_needles_ are the aboveground biomass for the stem, bark, branch, and needles (kg tree^−1^), respectively, D = DBH (cm), H = height (m). Subsequently, the total aboveground biomass (AGB_total_, kg tree^−1^) was calculated as the sum of the biomass of each individual tree component (Equation (6)) as follows:

(6) $${AGB}_{total}={AGB}_{stem}+{AGB}_{bark}+{AGB}_{branch}+{AGB}_{needles}$$

Belowground biomass (BGB) was calculated using [64] the allometric equation (Equation (7)) developed to estimate the weight of total root biomass for radiata pine as:

(7) $$\ln\left(BGB\right)=0.902*\ln{(AGB}_{total})-0.7368$$

where BGB = individual tree root biomass (kg tree^−1^).

The carbon stock of each individual tree biomass component was estimated by multiplying AGB_total_ and BGB by a carbon factor (CF), corresponding to the fraction of carbon of each biomass component. We used a CF of 0.48 according to what is recommended for [27].

Finally, individual plot stocking (NHA, trees ha^−1^), stand volume (VHA, m^3^ ha^−1^), basal area (BA, m^2^ ha^−1^), aboveground biomass carbon stock (AGBC, Mg ha^−1^), and belowground biomass carbon stock (BGBC, Mg ha^−1^) were estimated by adding and scaling to a hectare level the numbers of trees per plot, and individual tree volume, basal area, CAGB, and CBGB.

### 4.4. Soil Organic Carbon Sampling and Calculations

Organic carbon stock in the mineral soil (up to 1 m deep) estimates were obtained by considering a composite sample for 20 distributed systematically within each plot. Composite mineral soil samples were obtained with a soil auger at 3 depths: 0–20 cm, 20–40 cm, and 40–100 cm. A total of 90 composite mineral soil samples were obtained for all the evaluated sites in our study. Soil samples were air-dried at 30 °C, passed through a 2 mm sieve to remove all root and plant debris, and a 10 g subsample aliquot was taken and dried at 65 °C for 24 h and ground. A final 5 g aliquot was obtained for the determination of organic carbon using an IRMS analyzer (infrared mass spectroradiometer, SERCON Scientific Inc., Cheshire, UK).

At each site, a 1 m depth soil pit was dug to describe the visual soil profile characteristics and bulk density (BD) samples were obtained using a metal cylinder of 100 cm^3^ volume at 0–20, 20–40, and 40–100 cm depths. Each soil sample was stored and taken to the laboratory to be dried at 65 °C until constant in weight. A total of 30 soil bulk density samples were obtained for all the sampled sites. Additionally, for each sampled depth, a bulk 0.5 kg soil sample was taken for subsequent Boyoucos soil texture analyses, soil organic matter (O.M.) content determination [67], and to estimate the permanent wilting point and field capacity moisture retention curve points using a pressure plate apparatus (Soil Moisture Inc., Goleta, CA, USA). Soil water-holding capacity (SWHC) for each soil sample was estimated as the difference between the permanent wilting point (PWP) and field capacity point (FC). All soil laboratory analyses were carried out in the Soil, Water, and Forest Research Laboratory (LISAB) at the Faculty of Forest Sciences of the University of Concepción, Chile.

Organic carbon stock in the mineral soil (SOC_d_) at each depth (d_1_ = 0–20, d_2_ = 20–40, and d_3_ = 40–100 cm depth) was calculated using (Equation (8)):

(8) $${SOC}_{d}=C_{d}*D_{d}*{BD}_{d}*0.1$$

where SOC_d_ = soil organic carbon stock of depth d (Mg ha_−1_), C_d_ = soil organic carbon concentration by depth d (g kg_−1_), D_d_ = soil thickness of depth d (cm), BD_d_ = bulk density of depth d (g cm^−3^).

The total soil organic carbon stock (SOC) until 1 m of depth of each plot was calculated by summing the SOC_d_ estimates at each depth.

### 4.5. Forest Floor Carbon Sampling and Estimates

Forest floor carbon stock samples from organic horizons (Oi + Oe + Oa layers) were collected using a circular 25 cm in diameter cutting frame (490.9 cm^2^) at 10 systematically selected points within each plot. At each point, litter (pine needles in all stages of decomposition) and coarse woody debris were collected separately, for a total of 600 forest floor samples from all the evaluated plots. Each forest floor sample was stored in paper bags, labeled, and taken to the laboratory for drying at 65 °C until constant in weight. The forest floor and woody debris dry weight was recorded for each sample using a 0.01 g precision balance. After weighing, the 10 organic horizons samples and the 10 woody debris residue samples were composited and homogenized separately for each plot. Each pair of composite samples per plot was independently ground using a 250 μm sieve blade mill to obtain two independent 5 g subsample aliquots that were used for final carbon analysis. Analysis of all forest floor samples was carried out on a free ash basis to remove potential mineral soil contamination.

The carbon stock of the organic horizons and coarse woody debris was obtained by multiplying the biomass dry weight of each component at each sample point by the average plot carbon concentration of each component (%), expanded to a hectare level (Mg ha^−1^) and averaged for each plot. Total forest floor carbon stock (FFC) was finally estimated by summing the carbon stock of the organic horizons and the coarse woody debris estimated from each plot.

### 4.6. Data Analysis

The total carbon stock (TCS) for each plot was estimated as the sum of all evaluated components (AGBC, BGBC, SOC, and FFC). Data was evaluated for normality using a Shapiro–Wilk test (PROC UNIVARIATE) and Levene’s test for heteroscedasticity. Non-normally distributed data was transformed using a BoxCox transformation (PROC TRANSREG). Analysis of variance (ANOVA) for each above- and belowground component, total carbon stock, and for all stand inventory estimates was applied to evaluate the differences among sites (PROC REG). A simple *t*-test was used to compare volcanic sand and recent ash soil types (PROC TTEST). Linear and nonlinear regression analyses were used to evaluate the relationship between stand cumulative volume and SOC, forest floor, above- and belowground biomass, and site total carbon stocks. Models were compared using the coefficient of determination (r^2^), the Akaike information criteria (AIC), and RMSE values. Linear models were adjusted with and without dummy variables considering soil types, and by using a log-normal transformation of stand productivity estimates. Statistical analysis and regression models were fitted in SAS (SAS version 9.4, SAS Institute, Inc., Cary, NC, USA). All tests were considered significant at a level of α = 0.05.

## 5. Conclusions

In the evaluated adult radiata pine plantations, we found a significantly higher total carbon stock in recent ash soil compared to sandy soil sites. In both soil types, a substantial amount of carbon was found in the mineral soil up to a depth of 1 m, representing the main C pool in recent ash soil sites (59.6%) and the second-largest in sandy soil sites (41.5%). This emphasizes the importance of including assessments up to a 1 m depth for accurate C stock estimates. The forest floor represented the lowest carbon stock across all sites, with 2.9% for recent ash and 5.8% for sandy soil sites. However, it plays a crucial role in protecting the mineral soil, storing nutrients, and contributing organic matter to the mineral soil from aerial biomass. A strong and valuable relationship was observed between stand productivity at harvesting age and both the carbon stock in the mineral soil and the total carbon stock of the site. High carbon stocks in the mineral soil and differences in C pools according to soil type highlight the importance of considering soil type and stand productivity at mature stages (harvesting) to develop site-specific models that may improve estimates of forest site C stocks on intensively managed plantations.

## Figures and Tables

**Table 1:** Means of stand growth metrics by site. Lowercase letters indicate significant differences among site means (Tukey’s HSD test) considering an ANOVA analysis (*p* < 0.05). Capital letters indicate a significant difference (*p* < 0.05) between the means of recent ash and sandy soil sites (two-sample *t*-test analysis).

Soil Type	Site Code	NHA (Trees ha^−1^)	DBH (cm)	HT (m)	BA (m^2^ ha^−1^)	VOL (m^3^ ha^−1^)	MAI (m^3^ ha^−1^ yr^−1^)
Recent Ash	HI	353.3 ns	35.5 abc	31.3 bc	36.3 bc	387.6 bc	18.5 bc
	MO	420.0 ns	34.8 abc	37.1 a	41.1 abc	512.6 ab	25.6 ab
	RA	370.0 ns	41.1 a	30.5 c	44.4 abc	549.1 ab	25.0 ab
	SL	416.7 ns	33.8 bc	32.0 abc	39.2 abc	432.2 bc	20.6 abc
	ST	433.3 ns	38.2 a	37.0 a	51.9 a	646.3 a	28.1 a
Sandy	BR	493.3 ns	34.3 bc	27.4 c	46.1 abc	431.4 bc	20.5 abc
	CO	466.7 ns	36.9 ab	36.3 ab	51.3 a	625.4 a	27.2 a
	LC	520.0 ns	34.4 c	30.0 c	49.6 ab	517.5 ab	22.5 abc
	LR	503.3 ns	29.2 c	28.3 c	34.4 c	333.8 c	15.2 c
	MI	476.7 ns	35.6 c	30.2 c	45.5 abc	470.8 abc	21.4 abc
Recent Ash Soils (Mean)		398.7 B	36.7 A	33.6 A	42.6 ns	505.5 ns	23.5 ns
Sandy Soils (Mean)		492.0 A	34.1 B	30.5 B	45.4 ns	475.8 ns	21.4 ns

Site codes are = HI: Hijuelas, MO: Montpellier, RA: Ramadillas, SL: Santa Lidia, ST: Santa Teresa, BR: Brasil, CO: Coyanco, LC: Los Cuartos, LR: La Reforma, MI: Misque; NHA = stocking, DBH = diameter at breast height; HT = total height; BA = basal area; VOL = over bark stand volume; MAI = mean annual increment. ns = not significant.

**Table 2:** Average aboveground, belowground, total biomass, soil, forest floor, and total site carbon stock for all sites. Lowercase letters indicate significant differences among site means (Tukey’s HSD test) considering an ANOVA analysis within each soil type. Different capital letters indicate significant differences among soil type means.

Soil Type	Site Code	AGBC (Mg ha^−1^)	BGBC (Mg ha^−1^)	TBC (Mg ha^−1^)	SOC (Mg ha^−1^)	FFC (Mg ha^−1^)	TCS (Mg ha^−1^)
Recent Ash	HI	110.2 bc	27.9 cd	138.1 bc	244.6 ab	10.7 b	393.4 bc
	MO	147.1 abc	36.9 abcd	183.9 abc	296.8 a	13.7 ab	494.5 ab
	RA	151.6 ab	37.3 abcd	188.9 ab	312.3 a	11.8 b	512.9 ab
	SL	123.4 bc	31.3 cd	154.7 bc	255.0 ab	16.9 ab	426.6 abc
	ST	182.2 a	44.5 a	226.7 a	298.3 a	13.7 ab	538.7 a
Sandy	BR	123.9 bc	32.1 bcd	156.0 bc	188.9 bc	8.9 b	353.8 c
	CO	176.9 a	43.9 ab	220.8 a	172.7 bc	22.7 ab	416.2 abc
	LC	153.2 ab	39.3 abc	192.5 ab	135.2 cd	27.7 a	355.3 c
	LR	98.2 c	26.1 d	124.3 c	67.7 d	17.3 ab	209.4 d
	MI	134.3 abc	34.2 abcd	168.5 abc	135.1 cd	16.5 ab	320.2 cd
Recent Ash Soils (Mean)		142.9 ns	35.6 ns	178.5 ns	281.4 A	13.4 B	473.2 A
Sandy Soils (Mean)		137.3 ns	35.2 ns	172.4 ns	139.9 B	18.6 A	330.9 B

Site codes are = HI: Hijuelas, MO: Montpellier, RA: Ramadillas, SL: Santa Lidia, ST: Santa Teresa, BR: Brasil, CO: Coyanco, LC: Los Cuartos, LR: La Reforma, MI: Misque; AGBC = carbon stock in the aboveground biomass; BGBC = carbon stock in the belowground biomass; TBC = carbon stock in the total (aboveground + belowground) biomass; SOC = organic carbon stock in the mineral soil (up to 1 m deep); FFC = carbon stock in the forest floor; and TCS = total carbon stock of the stands. ns = not significant.

**Table 3:** Adjusted models between cumulative over bark stand volume at harvesting age (VOL) and soil organic carbon until 1 m depth (SOC). Model 1 represents the generalized model considering all evaluated sites, while Model 2 represents the dummy variable regression model.

Model	r^2^	RMSE	AIC	Parameter	Estimate	S.E.	*t*	*p*
(1) SOC = a + b × VOL	0.16	82.8	319.05	a	58.39	73.45	0.80	0.43
				b	0.31	0.14	2.16	0.041
(2) SOC = a + b × VOL + c × Soil (0 = Sand or 1 = Ash)	0.88	32.04		a	3.61	28.78	0.13	0.901
			268.66	b	0.26	0.06	4.55	<0.001
				c	148.95	12.45	11.96	<0.001

r^2^: coefficient of determination; RMSE: root mean square error; AIC: Akaike information criterion values; a, b, and c regression coefficients; S.E.: standard error; *t*: *t*-Statistics; *p*: *p*-values of regression coefficients.

**Table 4:** Adjusted models between cumulative over bark stand volume at harvesting age (VOL) and total carbon stock of stands (TCS). Model 1 represents the generalized model considering all evaluated sites, while Model 2 represents the dummy variable regression model.

Model	r^2^	RMSE	AIC	Parameter	Estimate	S.E.	*t*	*p*
(1) TCS = a + b × VOL	0.49	76.95	315.07	a	80.65	68.23	1.18	0.25
				b	0.66	0.13	4.91	0.041
(2) TCS = a + b × VOL + c × Soil (0 = Sand or 1 = Ash)	0.91	31.89		a	30.39	28.64	1.06	0.299
			268.41	b	0.61	0.06	10.87	<0.001
				c	136.66	12.39	11.03	<0.001

r^2^: coefficient of determination; RMSE: root mean square error; AIC: Akaike information criterion values; a, b, and c regression coefficients; S.E.: standard error; *t*: *t*-Statistics; *p*: *p*-values of regression coefficients.

**Table 5:** Site characteristics of selected locations that consider a range of productivity sites for contrasting sandy and recent ash soils.

Site Code	Soil Type	Soil Order ^a^	Soil Taxonomy ^a,b^	Soil Series ^b^	Drainage ^b^	MAT (°C)	MAP (mm)	Age (years)	MAI (m^3^ ha^−1^ yr^−1^)
Hijuelas (HI)	Recent Ash	Andisol	Medial, Mesic Typic Haploxerands	Santa Bárbara	Well-drained	11.0	1585	21	18.5
Montpellier (MO)	Recent Ash	Andisol	Medial, Mesic Typic Haploxerands	Santa Bárbara	Well-drained	11.4	1521	20	25.6
Ramadillas (RA)	Recent Ash	Andisol	Medial, Mesic Typic Haploxerands	Santa Bárbara	Well-drained	9.8	1492	22	25.0
Santa Lidia (SL)	Recent Ash	Andisol	Medial, Thermic Humic Haploxerands	Mayulermo	Well-drained	12.5	1207	21	20.6
Santa Teresa (ST)	Recent Ash	Andisol	Medial, Mesic Typic Haploxerands	Santa Bárbara	Well-drained	11.7	1460	23	28.1
Brasil (BR)	Sandy	Entisol	Mixed, Thermic Typic Xeropsamments	Arenales	Excessively drained	13.6	1131	21	20.5
Coyanco (CO)	Sandy	Entisol	Mixed, Thermic Typic Xeropsamments	Arenales	Excessively drained	13.7	1105	23	27.2
Los Cuartos (LC)	Sandy	Entisol	Mixed, Thermic Typic Xeropsamments	Coreo	Well-drained to excessively drained	13.4	1182	23	22.5
La Reforma (LR)	Sandy	Entisol	Mixed, Thermic Typic Xeropsamments	Coreo	Well-drained to excessively drained	13.6	1103	22	15.2
Misque (MI)	Sandy	Entisol	Mixed, Thermic Typic Xeropsamments	Arenales	Well-drained	13.7	1055	22	21.4

^a^ [39]. ^b^ [40,41]. MAT = mean annual temperature; MAP = mean annual precipitation; MAI = mean annual increment of stand volume (calculated as the MAI mean of three plots measured at each site).

**Table 6:** Mean physical and chemical soil properties values evaluated at 0–20, 20–40, and 40–100 cm depths from soil pits representing each selected site. Standard deviation for total carbon (C total) at each depth is indicated in parenthesis. Different capital letters indicate significant differences among soil type means for each depth.

Soil	Site	Depth	B.D.	C Total	O.M.	SWHC	Clay	Silt	Sand
Type	Code	(cm)	(gr cm^−3^)	%					
Recent Ash	HI	0–20	0.56	6.21 (0.49)	14.55	25.52	5.34	42.45	52.21
		20–40	0.61	4.02 (0.21)	12.43	22.75	2.76	32.51	64.73
		40–100	0.69	3.04 (0.63)	9.17	17.23	2.89	34.96	62.15
	MO	0–20	0.53	7.86 (0.29)	16.17	28.05	10.30	35.88	53.82
		20–40	0.59	5.36 (0.07)	13.14	17.38	2.67	29.82	67.51
		40–100	0.63	3.97 (0.17)	11.25	14.69	2.05	24.35	73.60
	RA	0–20	0.68	7.38 (0.28)	10.08	26.77	17.83	29.35	52.82
		20–40	0.71	5.56 (0.83)	10.00	22.20	20.15	26.85	53.00
		40–100	0.67	3.31 (0.99)	11.54	16.70	19.49	23.96	56.55
	SL	0–20	0.53	7.05 (1.01)	15.82	37.94	2.67	59.84	37.49
		20–40	0.64	4.76 (0.94)	10.91	36.03	7.13	65.34	27.53
		40–100	0.66	3.01 (0.40)	10.20	37.86	4.65	56.93	38.42
	ST	0–20	0.67	6.61 (0.72)	14.32	28.51	7.84	47.59	44.57
		20–40	0.65	4.72 (0.39)	12.11	18.24	4.18	33.71	62.11
		40–100	0.66	3.75 (0.35)	10.42	34.10	2.76	37.41	59.83
Sandy	BR	0–20	1.07	2.80 (0.36)	2.67	17.21	4.19	73.40	22.41
		20–40	1.09	1.74 (0.35)	2.21	19.84	4.05	52.92	43.02
		40–100	1.16	1.31 (0.64)	3.00	16.89	5.71	11.56	82.73
	CO	0–20	1.18	1.68 (0.21)	2.50	5.99	2.75	29.51	67.74
		20–40	1.19	1.55 (0.50)	2.93	6.92	2.66	19.51	77.82
		40–100	1.13	1.42 (0.15)	2.91	5.99	2.57	22.22	75.21
	LR	0–20	1.32	0.89 (0.56)	1.34	2.87	2.74	7.15	90.10
		20–40	1.54	0.62 (0.21)	1.69	3.53	2.59	4.78	92.63
		40–100	1.52	0.27 (0.23)	1.93	1.17	2.70	2.14	95.16
	LC	0–20	1.06	1.16 (0.69)	2.87	3.45	2.66	17.45	79.89
		20–40	1.19	1.03 (0.23)	2.62	5.69	5.22	1.92	92.85
		40–100	1.36	1.06 (0.43)	1.81	4.34	2.61	2.07	95.32
	MI	0–20	1.03	2.04 (0.52)	2.14	4.85	2.47	7.20	90.33
		20–40	1.35	1.20 (0.15)	2.08	6.74	0.26	9.46	90.28
		40–100	1.52	0.67 (0.17)	1.11	4.42	2.67	2.37	94.96
Recent Ash Soils (Mean)		0–20	0.59 B	7.02 A	14.19 A	29.36 A	8.79 A	43.02 A	48.18 B
		20–40	0.64 B	4.88 A	11.72 A	23.32 A	7.38 A	37.65 A	54.98 B
		40–100	0.66 B	3.42 A	10.52 A	24.12 A	6.37 A	35.52 A	58.11 B
Sandy Soils (Mean)		0–20	1.13 A	1.71 B	2.34 B	6.87 B	2.96 B	26.94 B	70.09 A
		20–40	1.27 A	1.23 B	2.31 B	8.54 B	2.95 B	17.72 B	79.32 A
		40–100	1.34 A	0.95 B	1.15 B	6.56 B	3.25 B	8.07 B	88.68 A

Site codes are = HI: Hijuelas, MO: Montpellier, RA: Ramadillas, SL: Santa Lidia, ST: Santa Teresa, BR: Brasil, CO: Coyanco, LC: Los Cuartos, LR: La Reforma, MI: Misque; B.D. = soil bulk density; C total = total carbon; SWHC = soil water-holding capacity; O.M. = organic matter.

## Data Availability

Restrictions apply to the datasets. The datasets presented in this article are not readily available because the data are part of an ongoing study.
